# Fur rubbing in *Plecturocebus cupreus* – an incidence of self-medication?

**DOI:** 10.5194/pb-9-7-2022

**Published:** 2022-05-17

**Authors:** Gurjit K. Theara, Juan Ruíz Macedo, Ricardo Zárate Gómez, Eckhard W. Heymann, Sofya Dolotovskaya

**Affiliations:** 1 Sociobiology/Anthropology, Georg-August-Universität Göttingen, Kellnerweg 6, 37077 Göttingen, Germany; 2 Verhaltensökologie & Soziobiologie, Deutsches Primatenzentrum – Leibniz-Institut für Primatenforschung, Kellnerweg 4, 37077 Göttingen, Germany; 3 Herbarium Amazonense, Esquina Pebas con Nanay, Iquitos, Peru; 4 Instituto de Investigaciones de la Amazonía Peruana (IIAP), Av. A. Quiñones km 2,5, Iquitos, Peru; 5 Laboratory of Comparative Ethology and Biocommunication, Severtsov Institute of Ecology and Evolution, Russian Academy of Sciences, Moscow, Russia

## Abstract

Fur rubbing, i.e. rubbing a substance or an object into the pelage, has been described in numerous Neotropical primate species, including species of titi monkeys, but it seems to be a rare behaviour. Here we describe a fur rubbing event in a wild coppery titi monkey (*Plecturocebus* *cupreus*) with *Psychotria* sp. (Rubiaceae) leaves observed and videotaped during a field study on vigilance behaviour between September–December 2019 in the Peruvian Amazon. Plants of the genus *Psychotria* contain a great diversity of secondary metabolites and are often used in traditional medicine. We suggest that the fur rubbing was an act of self-medication. This is the first record of fur rubbing in coppery titi monkeys in almost 4400 h of observation accumulated over more than 20 years.

## 1 Introduction

Nonhuman primates are known to manipulate non-food items they find in their surroundings and to ingest or apply them to their bodies. Such items include various plant parts, minerals, arthropods or man-made products (e.g. soap) (Baker, 1996; Bowler et al., 2015; Huffman, 1997; Pebsworth et al., 2021; Peckre et al., 2018). The ingestion or topical use of these items can serve as social communication or for self-medicative purposes, also known as zoopharmacognosy (Rounak et al., 2011). For example, in white-faced capuchin monkeys (*Cebus* *capucinus*) collective fur rubbing has been proposed as a way to socialise with other group members (Baker, 1996; Leca et al., 2007). In most cases, however, fur rubbing is performed individually and considered self-medication in a broad sense, i.e. a behaviour used for ectoparasite removal, repelling insects, treating skin infections and wounds, soothing or stimulating the skin or even as fur conditioning (Baker, 1996; Gibson, 1990; Mascaro et al., 2022; Westergaard and Fragaszy, 1987). Plant parts can be rubbed into the fur directly or after squeezing or chewing them (Huffman, 1997). Such plant part manipulation probably leads to the release of secondary compounds, which can then be applied onto the fur more easily when mixed with saliva (Huffman, 1997). Fur rubbing has been observed in a number of primate species, both in the wild and in captivity (e.g. Baker, 1996; Campbell, 2000; Laska et al., 2007; Morrogh-Bernard et al., 2017; Zito et al., 2003). Observations of fur rubbing in different species of titi monkeys are summarised in Table 1.

**Table 1:** A summary of fur rubbing with leaves observed in titi monkeys. NA: information not available.

Titi monkey species	Plant species (family)	Mode of leaf	Duration	Application on	Source
		handling	(s)	body area	
*Plecturocebus discolor*	*Tetrathylacium* sp. (Salicaceae)	chewing	NA	NA	Carrillo-Bilbao et al. (2005)
*Plecturocebus toppini* (formerly *Callicebus* *brunneus*)	Undetermined species from Annonaceae and Bignoniaceae	chewing	NA	abdominal area	Francis Bossuyt, per- sonal communication in Carillo-Bilbao et al. (2005)
*Callicebus coimbrai* and *Callicebus* *barbarabrownae*	*Bauhinia* sp. (Fabaceae)	squeezing with one or both hands	15–30	chest and abdominal area	Souza-Alves et al. (2018)
*Plecturocebus oenanthe* (formerly *Callicebus* *oenanthe*)	*Piper aduncum* (Piperaceae)	squeezing with both hands	300–900	chest and abdominal area	Huashuayo-Llamocca and Heymann (2017)
*Plecturocebus brunneus*	*Senna obtusifolia* (Fabaceae), *Piper tuberculatum* (Piperaceae)	chewing	NA	chest	Oliveira et al. (2020)
*Plecturocebus cupreus*	*Psychotria* sp. (Rubiaceae)	chewing	∼ 300	abdominal area	this report

Here we describe, for the first time, an event of fur rubbing by a coppery titi monkey (*Plecturocebus* *cupreus*) in the Peruvian Amazon.

## 2 Methods

The observation was made during a study at the Estación Biológica Quebrada Blanco (EBQB), a study site in the northeastern Peruvian Amazon (4 ${}^{\circ }$ 21 ${}^{\prime }$ S, 73 ${}^{\circ }$ 09 ${}^{\prime }$ W), some 90 km south southeast of Iquitos. For details of the study area see Heymann et al. (2019).

We observed four groups of coppery titi monkeys as part of a study on vigilance between September–December 2019. Group 4, in which we observed the fur rubbing behaviour, consisted of one adult male, one adult female and their subadult offspring. Observations were usually made from dawn (06:00 PET, Peru time​​​​​​​), when the group left their sleeping site, until almost dusk (17:00 PET), when they retreated to one. Throughout the study, we used instantaneous scan sampling of 2 min for standardised data collection and ad libitum sampling for recording unusual events (Altman, 1974).

## 3 Results

On the 12 November 2019 at 12:04 PET, the adult male of the group climbed down a liana and, stopping at the height of less than 2 m, ripped a handful of leaves from a *Psychotria* sp. (Rubiaceae) understory tree. Then he climbed back up the liana, sat down and started chewing on the leaves. Then he took the leaves out of his mouth and rubbed them slowly but firmly on his lower abdomen (Video supplement). He continued this action for about 5 min. Then he discarded the leaves and moved towards the group, which had remained < 5 m away during the event.

## 4 Discussion

We report here the first case of fur rubbing behaviour by a coppery titi monkey using the leaves of *Psychotria* sp. in the Peruvian Amazon. Based on the similarity in the mode of application of the chewed leaves on the body reported in other primates, we suggest a self-medicative function to the behaviour of the coppery titi monkey. In the absence of a species level plant identification, we cannot suggest a specific medicinal mechanism in titi monkeys.

Our interpretation of a self-medicative function is supported by the fact that species of *Psychotria* (one of the largest angiosperm genera with about 1650 species; Nepokroeff et al., 1999), which have been chemically analysed, include a diversity and considerable concentrations of secondary compounds. These compounds show cytotoxic, analgesic, antiviral and antifungal bioactivities (Calixto et al., 2016). This makes *Psychotria* popular in traditional medicine and as herbal medicinal products, used topically in cases of skin disorders, ocular disorders, to relieve fever, headaches and earaches (Calixto et al., 2016). The species identity could not be determined unambiguously, and therefore it is impossible to tell whether this specific plant is used in folk medicine. However, a number of *Psychotria* species are used in Amazonian (and Central and South American) ethnobotany (Benevides et al., 2005; Duke and Vasquez, 1994; see Table S1 in the Supplement). Therefore, it can be reasonably assumed that the plant used by the titi monkey could have some medical properties, too. Although *Psychotria* can include psychoactive tryptamines that show hallucinogenic activity (McKenna et al., 1984), this is unlikely to account for fur-rubbing, as oral uptake is required for this effect. Our on-field examination of the plant revealed the crushed leaves to have a cinnamon-like scent of ground leaves.

The observation has been made in November during the onset of the rainy season. Although the number of ectoparasites, like mosquitoes and ticks, might have been on the increase, *Psychotria* spp. are not known to have insect repellent properties. Restricting application to the abdominal part of the body is also not compatible with this explanation (Koyama et al., 2008; Simmen and Tarnaud, 2011).

Interestingly, this behaviour has been observed for the first time at the EBQB, despite almost 4400 h of observing coppery titi monkeys between 1997–2019. Obviously, it is a rare behaviour, which makes any interpretation difficult. Further detailed behavioural observations and identification of the plant species used are needed before the function of the behaviour can be conclusively determined. Only through the accumulation of this kind of anecdotal observations will it be possible to detect more general patterns that can lead to more conclusive interpretations.

## Supplement

10.5194/pb-9-7-2022-supplement The supplement related to this article is available online at: https://doi.org/10.5194/pb-9-7-2022-supplement.

## Data Availability

Data sharing is not applicable to this article as no datasets were generated or analysed during the current study.

## Video supplement

A video that documents the observed fur rubbing is available on (Theara et al., 2022).
