# Supplementary material for: Fur rubbing in Plecturocebus cupreus – an incidence of self-medication?
Source: Primate Biol. 2022 May 17;9(1):7–10. doi: 10.5194/pb-9-7-2022 (PMC9128366; doi:10.5194/pb-9-7-2022)
Supplement: The supplement related to this article is available online at: https://doi.org/10.5194/pb-9-7-2022-supplement. [file pb-9-7-supplement.pdf]

Supplement of Primate Biol., 9, 7–10, 2022
https://doi.org/10.5194/pb-9-7-2022-supplement
© Author(s) 2022. CC BY 4.0 License.

Primate Biology
Open Access

Supplement of

Fur rubbing in *Plecturocebus cupreus* – an incidence of self-medication?

Gurjit K. Theara et al.

Correspondence to: Gurjit K. Theara (gurjit.theara@yahoo.de)

The copyright of individual parts of the supplement might differ from the article licence.

Table S1

Supplementary information

References for research on bioactive compounds and ethnobotany of Neotropical species of the genus *Psychotria*. This list is not exhaustive, but demonstrates that bioactive compounds are widespread in the genus and that various species are used in traditional medicine. This makes it plausible that the *Psychotria* leaves used by *Plecturocebus cupreus* might also include such compounds.

**Reference**	***Psychotria* species**
Agripino DG, Lima MEL, Silva MRd, Meda CI, Bolzani VdS, Cordeiro I, Young MCM, Moreno PRH (2004)<br>Screening of Brazilian plants for antimicrobial and DNA-damaging activities: I. Atlantic rain forest. Ecological Station Juréia-Itatins.<br>*Biota Neotropica* 4:1-15	*P. mapoureoides*
Benevides PJC, Young MCM, Bolzani V de S (2005)<br>Biological activities of constituents from *Psychotria spectabilis*.<br>*Pharmaceutical Biology* 42:565-569	*P. spectabilis*
Bussmann RW, Sharon D (2018)<br>Medicinal plants of the Andes and the Amazon - The magic and medicinal flora of Northern Peru.<br>*Ethnobotany Research and Applications* 15:1-292	*P.* sp.
Calixto NO, Pinto MEF, Ramalho SD, Burger M, Bobey AF, Young MCM, Bolzani VS, Pinto AC (2016)<br>The genus *Psychotria*: phytochemistry, chemotaxonomy, ethnopharmacology and biological properties.<br>*Journal of the Brazilian Chemical Society* 27:1355-1378	numerous species
Cragg GM, Newman DJ, Yang SS (2006)<br>Natural product extracts of plant and marine origin having antileukemia potential. The NCI experience.<br>*Journal* of *Natural Products* 69:488-498	*P.* sp.
da Silva Moraes TM, de Araújo MH, Bernardes NR, de Oliveira DB, Lasunskaia EB, Muzitano MF, Da Cunha M (2011)<br>Antimycobacterial activity and alkaloid prospection of *Psychotria* species (Rubiaceae) from the Brazilian Atlantic Rainforest.<br>*Planta Medica* 77:964-970	*P. capitata*<br>*P. glaziovii*<br>*P. leiocarpa*<br>*P. nuda*<br>*P. pubigera*<br>*P. racemosa*<br>*P. ruelliifolia*<br>*P. stachyoides*

	P. suterela [sic]<br>*P. vellosiana*
do Nascimento NC, Fragoso V, Moura DJ, e Silva ACR, Fett-Neto AG, Saffi J (2007)<br>Antioxidant and antimutagenic effects of the crude foliar extract and the alkaloid brachycerine of *Psychotria brachyceras*.<br>*Environmental and Molecular Mutagenesis* 48:728-734	*P. brachyceras*
Duke JA, Vasquez R (1994)<br>Amazonian ethnobotanical dictionary.<br>Boca Raton, CRC Press	*P. acuminata*<br>*P. alba*<br>*P. carthaginensis*<br>*P. deflexa*<br>*P. marginata*<br>*P. poeppigiana*<br>*P. stenostachya*<br>*P. viridis*
Elisabetsky E, Castilhos ZC (1990)<br>Plants used as analgesics by Amazonian caboclos as a basis for selecting plants for investigation.<br>*International Journal of Crude* Drug *Research* 28:309-320	*P. colorata*
Formagio ASN, Volobuff CRF, Santiago M, Cardoso CAL, Vieira MDC, Valdevina Pereira Z (2014)<br>Evaluation of antioxidant activity, total flavonoids, tannins and phenolic compounds in *Psychotria* leaf extracts.<br>*Antioxidants* 3:745-757	*P. capillacea*<br>*P. carthagenensis*<br>*P. deflexa*<br>*P. leiocarpa*
Gupta MP, Solis PN, Calderón AI, Guinneau-Sinclair F, Correa M, Galdames C, Guerra C, Espinosa A, Alvenda GI, Robles G (2005)<br>Medical ethnobotany of the Teribes of Bocas del Toro, Panama.<br>*Journal of Ethnopharmacology* 96:389-401	*P. emetic*<br>*P. psychotriifolia*
Henriques AT, Lopes SO, Paranhos JT, Gregianini TS, Fett-Neto AG, Schripsema J, Von Poser GL (2004)<br>N, β-D-Glucopyranosyl vincosamide, a light regulated indole alkaloid from the shoots of *Psychotria leiocarpa*.<br>*Phytochemistry* 65:449-454	*P. leiocarpa*
Joly LG, Guerra S, Septimo R, Solis PN, Correa M, Gupta M, Levy S, Sandberg F (1987)<br>Ethnobotanical inventory of medicinal plants used by the Guaymi Indians in Western Panama. Part I.<br>*Journal of Ethnopharmacology* 20:145-171	*P. uliginosa*
Kerber VA, Passos CS, Verli H, Fett-Neto AG, Quirion J, Henriques AT (2008)<br>Psychollatine, a glucosidic monoterpene indole alkaloid from *Psychotria umbellata*.<br>*Journal of Natural Products* 71:697-700	*P. umbellata*

Leal MB, Elisabetsky E (1996)<br>Opioid-like activity of *Psychotria brachypoda*.<br>*International Journal of Pharmacognosy* 34:267-272	*P. brachypoda*
Luziatelli G, Sørensen M, Theilade I, Mølgaard P (2010)<br>Asháninka medicinal plants: a case study from the native community of Bajo Quimiriki, Junín, Peru.<br>*Journal of Ethnobiology and Ethnomedicine* 6:21	*P. poeppigiana*
McKenna DJ, Towers GHN, Abbott F (1984)<br>Monoamine oxidase inhibitors in South American hallucinogenic plants: tryptamine and β-carboline constituents of ayahuasca.<br>*Journal of Ethnopharmacology* 10:195-223	*P. carthagenensis*<br>*P. viridis*
Moura LTS, Maruo VM (2014)<br>Aspectos farmacológicos e toxicológicos de *Psychotria colorata* revisão.<br>*Revista Científica de Medicina Veterinaria* 12:1-16	*P. colorata*
Nomura T, Quesada AL, Kutchan TM (2008)<br>The new β-D-glucosidase in terpenoid-isoquinoline alkaloid biosynthesis in *Psychotria ipecacuanha*.<br>*Journal of Biological Chemistry* 283:34650-34659	*P. ipecacuanha*
Paniagua Zambrana NY, Bussmann RW, Hart RE, Moya Huanca AL, Ortiz Soria G, Ortiz Vaca M, Ortiz Álvarez D, Soria Morán J, Soria Morán M, Chávez S, Chávez Moreno B, Chávez Moreno G, Roca O, Siripi E (2017)<br>Traditional knowledge hiding in plain sight – twenty-first century ethnobotany of the Chácobo in Beni, Bolivia.<br>*Journal of Ethnobiology and Ethnomedicine* 13:57	*P. lupulina*<br>*P. prunifolia*
Pimenta ATA, Uchôa DEA, Braz-Filho R, Silveira ER, Lima MAS (2011) Alkaloid and other chemical constituents from *Psychotria stachyoides* Benth. *Journal of the Brazilian Chemical Society* 22:2216-2219	*P stachyoides*
Rosales-López C, Muñoz-Arrieta R, Abdelnour-Esquivel A (2020)<br>Emetine and cephaeline content in plants of *Psychotria ipecacuanha* in Costa Rica.<br>*Revista Colombiana de Química* 49:18-22	*P. ipecacuanha*
Sanz-Biset J, Campos-de-la-Cruz J, Epiquién-Rivera MA, Cañigueral S (2009)<br>A first survey on the medicinal plants of the Chazuta valley (Peruvian Amazon).<br>*Journal of Ethnopharmacology* 122:333-362	*P. alba*<br>*P. carthagensis*<br>*P. ernestii*<br>*P. viridis*
Verotta L, Pilati T, Tato M, Elisabetsky E, Amador TA, Nunes DS (1998)<br>Pyrrolidinoindoline alkaloids from *Psychotria colorata*.<br>*Journal of Natural Products* 61:392-396	*P. colorata*
